# Supplementary material for: Contextual Interference Effect Is Independent of Retroactive Inhibition but Variable Practice Is Not Always Beneficial
Source: Front Hum Neurosci. 2019 May 31;13:165. doi: 10.3389/fnhum.2019.00165 (PMC6557302; doi:10.3389/fnhum.2019.00165)
Supplement: Supplementary file 1 [file Data_Sheet_1.pdf]

Supplementary material

"Contextual interference effect is independent of retroactive inhibition but variable practice is not always beneficial"

Benjamin Thürer[1,2]*, Sarah Gedemer[2], Anne Focke[2], Thorsten Stein[2]
1: Brain Signaling Group, Institute of Basic Medical Sciences, Department of Molecular Medicine, University of Oslo, Oslo, Norway
2: BioMotion Center, Institute of Sports and Sports Science, Karlsruhe Institute of Technology, Karlsruhe, Germany

Correspondence:
Dr. Benjamin Thürer
benjamin.thurer@medisin.uio.no

Table S1: Overview of the force field magnitudes on the subject level.

Group	*N* =	Practice			Posttest			Transfer
		**1. Block**	**2. Block**	**3. Block**	**1. Block**	**2. Block**	**3. Block**	
**BM**	*2*	8	15	22	22	15	8	22
	2	22	8	15	15	8	22	15
	2	15	22	8	8	22	15	8
	2	8	22	15	15	22	8	15
	2	15	8	22	22	8	15	22
	2	22	15	8	8	15	22	8
**BU**	*2*	8	15	22	8	15	22	8
	2	22	8	15	22	8	15	22
	2	15	22	8	15	22	8	15
	2	8	22	15	8	22	15	8
	2	15	8	22	15	8	22	15
	2	22	15	8	22	15	8	22
**R**	*6*	random	random	random	15	8	22	15
	6	random	random	random	15	22	8	15
**C**	*2*	8	8	8	8	15	22	8
	2	15	15	15	15	8	22	15
	2	22	22	22	22	15	8	22
	2	8	8	8	8	22	15	8
	2	15	15	15	15	22	8	15
	2	22	22	22	22	8	15	22

Overview table about all force field magnitudes and the counterbalancing within and across groups. All values represent the force-field magnitudes in Ns/m; *N*: number of subjects; random: random order of force field magnitudes; BM: Blocked-Matched group; BU: Blocked-Unmatched group; R: Random group; C: Constant group.

Bayes statistics

It is widely accepted that p-values are not a good marker for potential "findings" in research due to different ways to bias p-values and due to false interpretations (e.g. Nuzzo, 2014). Therefore, besides using classical inferential statistics, we also used Bayesian statistics to test our findings. Therefore, Cauchy prior width was set to Cohen's $d = 0.8$ for the t-tests and $r = 0.5$ for ANOVAs so that 50 % of the prior mass falls into and the other 50 % exceeds the range of a high effect size according to Cohen (1988). We checked each statistical analysis for the Bayes model error, which did not exceed 5 %. The reported Bayes Factor (BF_{10}), therefore, represents a ratio how much the alternative hypothesis (H1) is more likely than the null hypothesis (H0) under consideration of a high effect size (Rouder et al., 2012).

Table S2: **Results of the Bayesian statistics**

		**BM vs. BU** *(BF_{10})*	**BM vs. R** *(BF_{10})*	**BU vs. R** *(BF_{10})*
**Adaptation**	Time	7.02e+11	6.40e+8	2.94e+7
	Group	0.67	0.34	0.43
	Time*Group	0.84	8.89	0.93
**Consolidation** (First 6 Trials)	Time	6385.13	472.67	148.26
	Group	0.47	1.74	0.49
	Time*Group	0.46	0.42	0.69
**Consolidation** (All trials)	Time	3.72e+5	12.07	9.76
	Group	0.44	1.74	0.81
	Time*Group	0.39	2.46	2.58
**Transfer**	Time	1.73e+8	5.38e+8	9.83e+7
	Group	0.33	0.44	0.47
	Time*Group	0.39	0.58	0.49

Testing for Adaptation, mixed model ANOVA with factors time (Practice FT, Practice LT) and group (BM, BU; BM, R; BU, R) was used. For Consolidation purposes, the factor time of the ANOVAs contained either Practice LT and Posttest FT or Practice LT and Posttest ALL. Testing for Transfer, ANOVA used Practice LT and Transfer FT for the factor time.

Figure S1: Deeper investigation of the benefits after random practice.

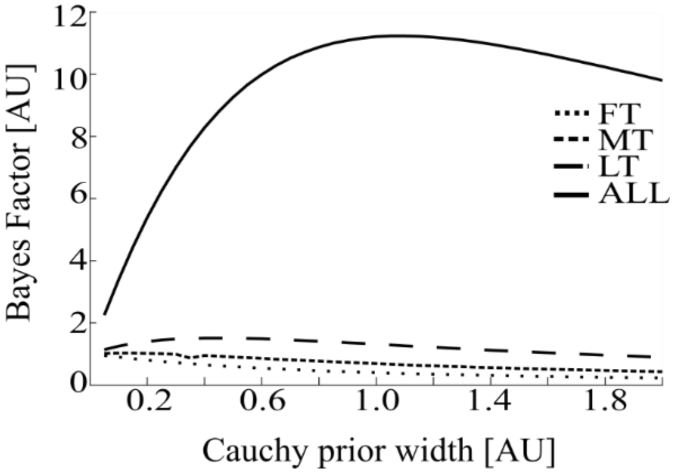

Bayesian statistic factor (BF_{10}) in dependency of the Cauchy prior width. High BF_{10} values indicate how much the alternative hypothesis (R unequal C) is more likely than the null hypothesis (R equal C). This figure shows that the Random group had a better mean memory consolidation across all force field magnitudes, with this effect most pronounced predicting high effect sizes.

Calculation of force field prediction

It is possible that group differences during Practice arise from the random practice schedule and that the internal model within the Random group for a given force field magnitude (here 15 Ns/m because this is the Posttest magnitude) is similar to that of the Blocked group (with individual force field magnitude dependent on the last practiced force field). In other words, it is possible that Blocked and Random groups do not deviate from each other in their Posttest performance because their internal model was equally adapted after Practice. If this is correct, there should be no difference between Random and Blocked subjects in their force-field prediction. Analyses of error-clamp trials (trials in which force channels guide the subject's movement directly towards the target) should lead to similar force-field compensation factors (factor describing the force field prediction based on the amount of force against the channel walls) between groups after the Practice schedule. Therefore, we calculated the force field compensation factor (see Thürer & Weber et al. 2018, for more details) for each individual subject in the 6 error-clamp trials after Practice. In each error-clamp trial, we quantified the force field compensation factor by comparing the measured with the ideal perpendicular force profile using linear regression and averaged those across the 6 error clamp trials. Regression analysis was done by using the polyfit function in MATLAB with a degree of 1. The first coefficient of the linear component was used for computing the force field compensation. As participants did not receive error-feedback during these trials, this parameter mainly reflected force field predictions, and thus, feedforward mechanisms (all scripts are available by the first-author or at: https://github.com/benjaminthuerer/Force-Field-Learning_analysis).

Mann-Whitney U tests between the Random and Blocked groups were, indeed, not significant but showed a low p-value combined with a mid-to-high effect size (U = 195, p = 0.09, d = 0.708, r = 0.354; with d representing Cohen's d and r representing rank biserial correlation). This indicates that the confounding effect, suggested here, plays a role but also it indicates that force field prediction differed between groups at the end of Practice.

Figure S2: Differences between Blocked and Random groups in force field prediction.

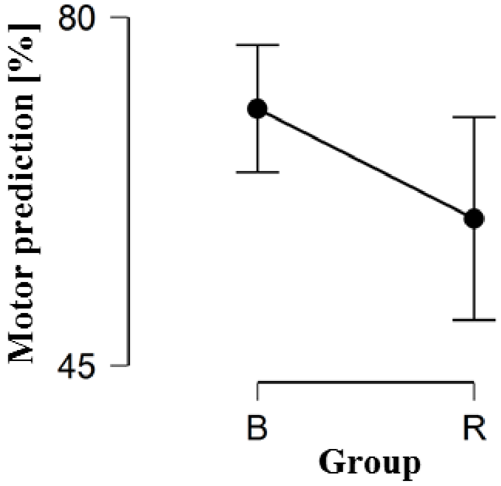

Mean motor prediction with 95% Confidence interval for Blocked (B) and Random (R) subjects.

References

Cohen J (1988). *Statistical power analysis for the behavioral sciences*. New York: Academic Press.

Nuzzo R (2014). Scientific method: statistical errors. *Nature News*, 506(7487), 150.

Rouder JN, Morey RD, Speckman PL, Province JM (2012). Default Bayes factors for ANOVA designs. *Journal of Mathematical Psychology*, 56(5), 356-374.
